# The Complete Mitochondrial Genome of the Pink Stem Borer, *Sesamia inferens*, in Comparison with Four Other Noctuid Moths

**DOI:** 10.3390/ijms130810236

**Published:** 2012-08-16

**Authors:** Huan-Na Chai, Yu-Zhou Du

**Affiliations:** Institute of Applied Entomology, Yangzhou University, Yangzhou 225009, China; E-Mails: chaihuanna@126.com

**Keywords:** *Sesamia inferens*, mitochondrial genome, lepidoptera, Noctuidae, phylogenetic relationship

## Abstract

The complete 15,413-bp mitochondrial genome (mitogenome) of *Sesamia inferens* (Walker) (Lepidoptera: Noctuidae) was sequenced and compared with those of four other noctuid moths. All of the mitogenomes analyzed displayed similar characteristics with respect to gene content, genome organization, nucleotide comparison, and codon usages. Twelve-one protein-coding genes (PCGs) utilized the standard ATN, but the *cox1* gene used CGA as the initiation codon; *cox1*, *cox2*, and *nad4* genes had the truncated termination codon T in the *S. inferens* mitogenome. All of the tRNA genes had typical cloverleaf secondary structures except for *trnS1(AGN)*, in which the dihydrouridine (DHU) arm did not form a stable stem-loop structure. Both the secondary structures of *rrnL* and *rrnS* genes inferred from the *S. inferens* mitogenome closely resembled those of other noctuid moths. In the A+T-rich region, the conserved motif “ATAGA” followed by a long T-stretch was observed in all noctuid moths, but other specific tandem-repeat elements were more variable. Additionally, the *S. inferens* mitogenome contained a potential stem-loop structure, a duplicated 17-bp repeat element, a decuplicated segment, and a microsatellite “(AT)_7_”, without a poly-A element upstream of the *trnM* in the A+T-rich region. Finally, the phylogenetic relationships were reconstructed based on amino acid sequences of mitochondrial 13 PCGs, which support the traditional morphologically based view of relationships within the Noctuidae.

## 1. Introduction

The pink stem borer, *Sesamia inferens* (Walker), is an important rice pest that is widely distributed in China, Japan, India and other countries, and causes severe damage to rice crops in rice planting areas [1–3]. Since 1990, with the expansion of hybrid rice planting areas and changes in climate and rice cultivation systems, the *S. inferens* population has been gradually increasing in China, and has become an important rice pest next to *Chilo suppressalis* (Walker), especially in the Lower–Middle Reaches of the Yangze River [3]. At present, the mitochondrial genomes (mitogenomes) from more than 393 species of arthropods have been completely or partially determined, including 241 species of insects. However, only 36 mitogenomes from six lepidopteran superfamilies have been sequenced, with seven species of Bombycoidea, one species of Geometroidea, 14 species of Papilionoidea, six species of Pyraloidea, three species of Tortricoidea, and five species of Noctuoidea, including *Helicoverpa armigera*, *Hyphantria cunea*, *Lymantria dispar*, *Ochrogaster lunifer*, and *S. inferens* [4–7].

The insect mitogenome is normally a closed-circular duplex molecule, ranging from 14 to 20 kb in length. The mitogenome usually contains 13 protein-coding genes (PCGs: *atp6*, *atp8*, *cox1*, *cox2*, *cox3*, *cob*, *nad1*, *nad2*, *nad3*, *nad4*, *nad5*, *nad6*, and *nad4L*), large and small ribosomal RNA genes (*rrnL* and *rrnS*), 22 tRNA genes, which are involved in energy production, electron transport and oxidative phosphorylation [8,9]. In addition, at least one segment of the most variable A+T-rich region is present in the insect mitogenome, including initiation sites for the transcription and replication of the genome [10,11].

Compared with the nuclear genome, the animal mitogenome—except for lice [12]—usually has a smaller size, a stable and relatively short circular structure. In addition, it has other characteristics such as a higher rate of base substitution and a presumed lack of intermolecular recombination. Mitogenome has been widely used as an informative molecular marker to reveal fundamental information for phylogenetic inference, the identification of species, phylogeography, the analysis of population structure and dynamics, and molecular evolution at the genomic level. This has been especially true for the past several decades, especially since the human mitogenome was sequenced [13]. More and more insect mitogenomes will increase the richness of information for phylogenetic analyses and evolutionary biology.

In this study, we report the completed mitogenome of *S. inferens* and provide a thorough description of its genomic features, including gene order, nucleotide composition of PCGs, secondary structures of tRNA and rRNA genes, and A+T-rich region. In addition, we compare the *S. inferens* mitogenome with those of four other noctuid moths, which can provide further insights into the relationships among Noctuidae species. Detailed genetic information on this important rice pest may help in the development of methods for its control or prevention.

## 2. Results and Discussion

### 2.1. Genome Structure, Organization and Composition

**Figure 1:**
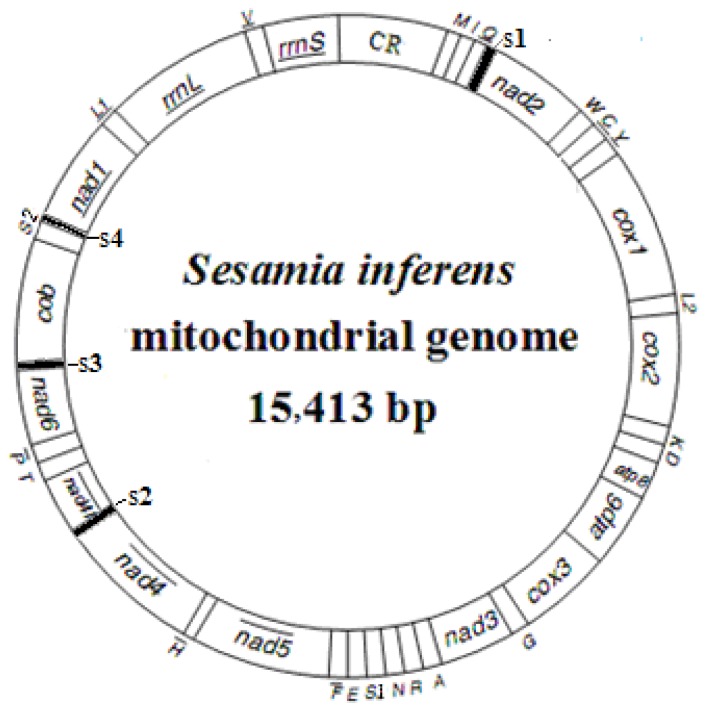
A map of the mitogenome of *Sesamia inferens*. Transfer RNA genes are designated by single-letter amino acid codes. CR represents the A+T-rich region. The gene name without underline indicates the direction of transcription from left to right, and with underline indicates right to left. s1–s4, intergenic spacers.

The complete mitogenome of *S. inferens* was found to be a 15,413 bp long circular molecule, and its sequence was deposited into GenBank (Accession number: JN039362) (Figure 1 and Table 1). The size was shorter than that of *H. cunea* (15,481 bp), *L. dispar* (15,569 bp), and *O. lunifer* (15,593 bp) [4,5,7]. All of the 37 typical animal mitochondrial genes (13 PCGs, 22 tRNA and 2 rRNA genes) were present, and the gene order was the same as in other sequenced noctuid moths, in an order of *trnM*, *trnI*, and *trnQ*. The position of the *trnM* gene was usually translocated to the 5′ upstream position of *trnI* in the lepidopteran species, which was different from those of other order species [14]. This indicated that the mitochondrial gene arrangement in lepidopteran species evolved independently after splitting from its stem lineage [15]. In addition, a total of 257 bp of intergenic spacer sequences were present in 18 locations with the exception of the A+T-rich region, and a total of 25 bp of overlapping nucleotides was scattered over six locations in the *S. inferens* mitogenome.

The nucleotide composition of the *S. inferens* mitogenome was also biased toward AT nucleotides (80.3%), which was slightly higher than those of *L. dispar* (79.9%) and *O. lunifer* (77.8%), but was slightly lower than those of *H. armigera* (81.0%) and *H. cunea* (80.4%) [4–7] (Table 2). The AT skew in the *S. inferens* mitogenome was slightly negative (−0.001), indicating the occurrence of more Ts than As. This value was different from those of other Noctuidae species, such as *H. armigera* (0.002), *H. cunea* (0.010), *L. dispar* (0.016), *O. lunifer* (0.030) [4–7]. Meanwhile, the GC skew in the *S. inferens* mitogenome was negative (−0.228), demonstrating the occurrence of more Cs than Gs. This was also true for *H. armigera* (−0.184), *H. cunea* (−0.230), *L. dispar* (−0.247), *O. lunifer* (−0.318) [4–7].

### 2.2. Protein-Coding Genes

The mitochondrial sequence of *S. inferens*, as in most insects, contained 13 PCGs (Table 2). Most of the PCGs utilized typical ATN start codons (ATG, ATT, and ATC), among which six genes started with codon ATG (*atp6*, *cox3*, *nad4*, *nad4L*, *cob*, and *nad1*), five with ATT (*nad2*, *cox2*, *atp8*, *nad3*, and *nad5*), and the *nad6* gene with ATC. However, the *cox1* gene in the *S. inferens* mitogenome had the start codon CGA as observed in most other lepidopteran species sequenced to date [16]. Notably, the start codon of the *cox1* gene is usually found to use nonstandard putative codons, as previously reported in arthropod species [17–20].

The conventional termination codon TAA, likely resulting from post-transcriptional polyadenylation [21], was observed in 10 PCGs. However, the *cox1*, *cox2*, and *nad4* genes utilized truncated termination codons, which are commonly observed in lepidopteran species.

The average AT content of the 13 PCGs in the *S. inferens* mitogenome was 78.6%, with a negative AT skew of −0.148 and a slightly positive GC skew of 0.037 (Table 2). The AT and GC skews were similar to those of *H. armigera* (−0.139, 0.029), *H. cunea* (−0.146, 0.023), and *L. dispar* (−0.148, 0.013), indicating that the contents of T and G were higher than those of A and C, respectively [5–7]. It is to be noted that both the AT skew (−0.141) and the GC skew (−0.004) were slightly negative in the 13 PCGs of *O. lunifer* [4]. Also, the AT contents at different codon positions was analyzed. In the *S. inferens* mitogenome, the AT contents was 87.7%, 72.9% and 75.2% at the first, second and third codon positions, respectively, with the highest AT contents at the first codon position, which was similar to the result from *L. dispar* mitogenome (90.2%, 72.9%, 70.1%), but was different from that in *H. armigera* (70.2%, 94.1%, 73.8%), *H. cunea* (73.2%, 70.3%, 92.0%) and *O. lunifer* mitogenomes (72.0%, 70.0%, 85.1%) [4–7] (Table 2).

**Figure 2:**
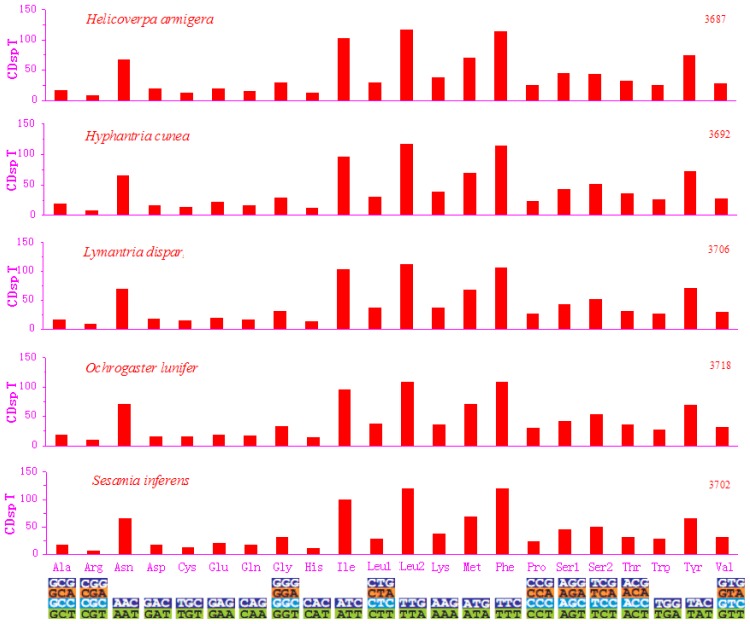
Codon distributions in five noctuid moths’ mitogenomes as indicated. The total number of codons per thousand codons (designated as CDsp T) is shown on the *y*-axis and the codon families on the *x*-axis.

**Figure 3:**
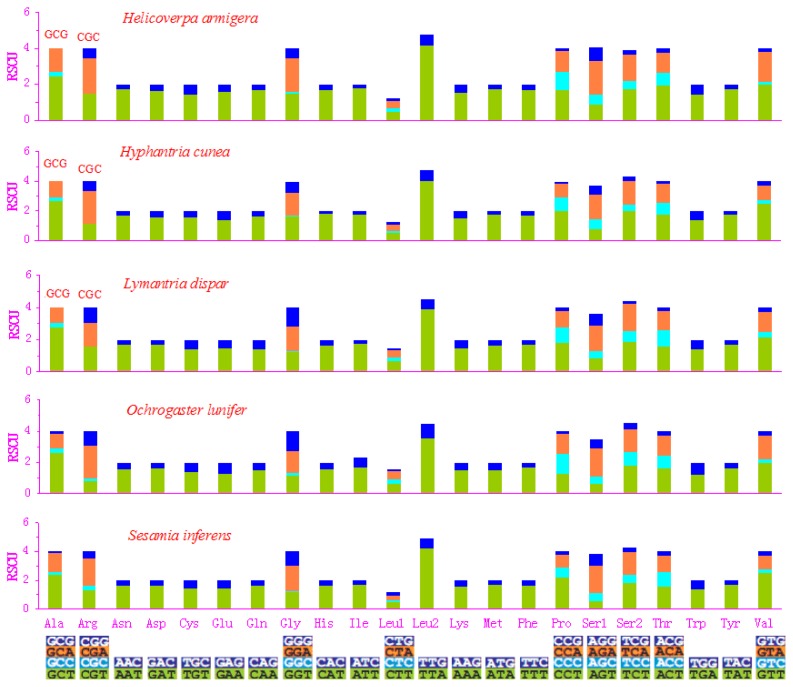
The relative synonymous codon usage (RSCU) in five noctuid moths’ mitogenomes as a function of codon families. Red codons indicate that the codons are missing in the mitogenome.

Excluding the start and termination codons, the 13 PCGs in the *S. inferens* mitogenome consisted of 3702 codons in total, consistent with the observations in four other noctuid moths, which ranged from 3687 in the *H. armigera* to 3718 codons in the *O. lunifer* mitogenome [4–7] (Figure 2). The codon families exhibited a very similar behavior among the five species (Figures 2 and 3). There were at least six codon families with at least 50 codons (CDs) (Asn, Ile, Leu2, Met, Phe, Tyr), and two families with at least 100 CDs per thousand CDs (Leu2 and Phe) as observed in five insects. In addition, the AT-rich CDs favor synonymous CDs with a lower AT content, as revealed by the Relative Synonymous Codon Usage (RSCU) results, and exemplified by the Leu2 family, in which the TTA codon accounted for the large majority of CDs (Figure 3). The codon families with high CDs had A and T predominantly in the third position, which might reflect selection for optimal tRNA usage, genome bias, and the speed and efficacy of genome/DNA repair [22].

### 2.3. Transfer RNA Genes

**Figure 4:**
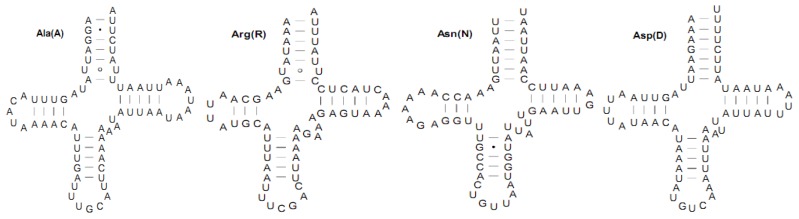

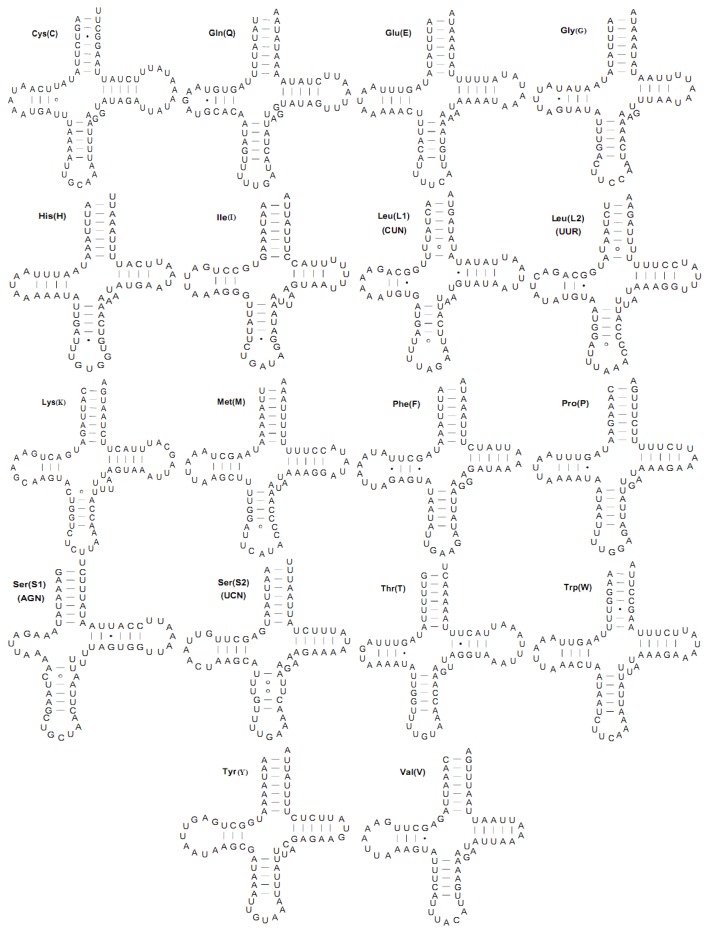

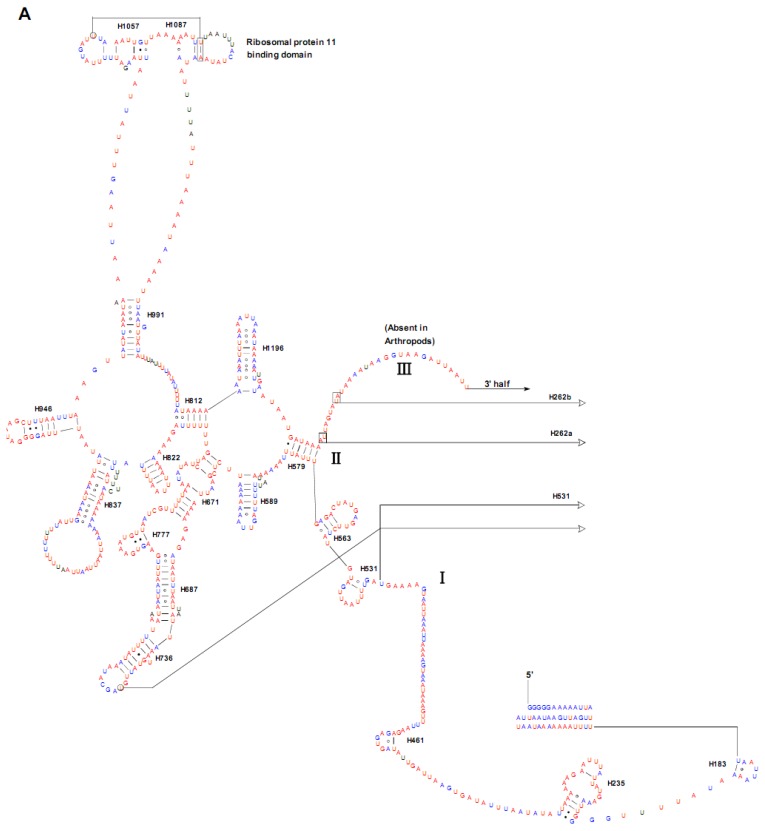
Inferred secondary structures for 22 typical tRNAs of the *Sesamia inferens* mitogenome. The tRNAs are labeled with the abbreviations of their corresponding amino acids. Base-pairing is indicated as follows: Watson–Crick pairs by lines, wobble GU pairs by dots and mismatched pairs by circles.

The mitogenome of *S. inferens* had the characteristic 22 tRNAs sets interspersed by rRNAs or PCGs, with an AT content of 81.5% and a total of 1478 bp in size ranging from 64 to 73 bp/tRNA. All tRNA genes had the typical cloverleaf secondary structures except for the *trnS1(AGN)* gene, in which a stable stem-loop structure of the dihydrouridine (DHU) arm was missing (Figure 4), as observed in many metazoan mitogenomes [23–25]. Eight tRNAs were encoded by the L-strand and the remaining 14 by the H-strand, which was identical in all the species. All tRNA genes usually contained a 7-bp amino acid acceptor (AA) stem, where most nucleotide substitutions were compensatory. The anticodon (AC) stem and the loop (7 bp) were both conserved in all tRNAs, whereas two U–U pairs were usually located at the second and third couplets in the anticodon stem of *trnS2(UCN)*. The length of DHU was 3–4 bp, except for *trnS1(AGN)*. The TΨC arm was usually 3–6 bp in length. Except for *trnD*, *trnE*, and *trnY*, a total of 29 unmatched base pairs were detected in the *S. inferens* tRNAs, but 18 of them were G-U pairs, which form a weak bond in the tRNAs, and are well-known non-canonical pairs in tRNA secondary structures [26]. The remaining 11 were mismatched base pairs including seven U-U, one C-U, and three A-C pairs. Notably, the numbers of mismatches varied in different noctuid moths, with 4, 7, 23 and 10 mismatches in *H. armigera*, *H. cunea*, *L. dispar*, and *O. lunifer*, respectively [4–7].

### 2.4. Ribosomal RNA Genes

As in other mitochondrial sequences from Insecta species, there were two rRNAs in *S. inferens* with a total length of 2169 bp and an AT content of 84.1%. The large ribosomal gene (*rrnL*) had a length of 1385 bp, located between *trnL1(CUN)* and *trnV*, whereas the small one (*rrnS*) had a length of 784 bp between *trnV* and the A+T-rich region. The gene sizes and locations were typical as in other noctuid moths’ mitogenomes. Both tRNA and rRNA genes of Noctuidae species predominantly contained A and T [4–7] (Table 2). Furthermore, both the AT and GC skews were slightly positive in the tRNA and rRNA genes, consistent with the results in *H. armigera* and *H. cunea* mitogenomes [5,6].

**Figure 5:**
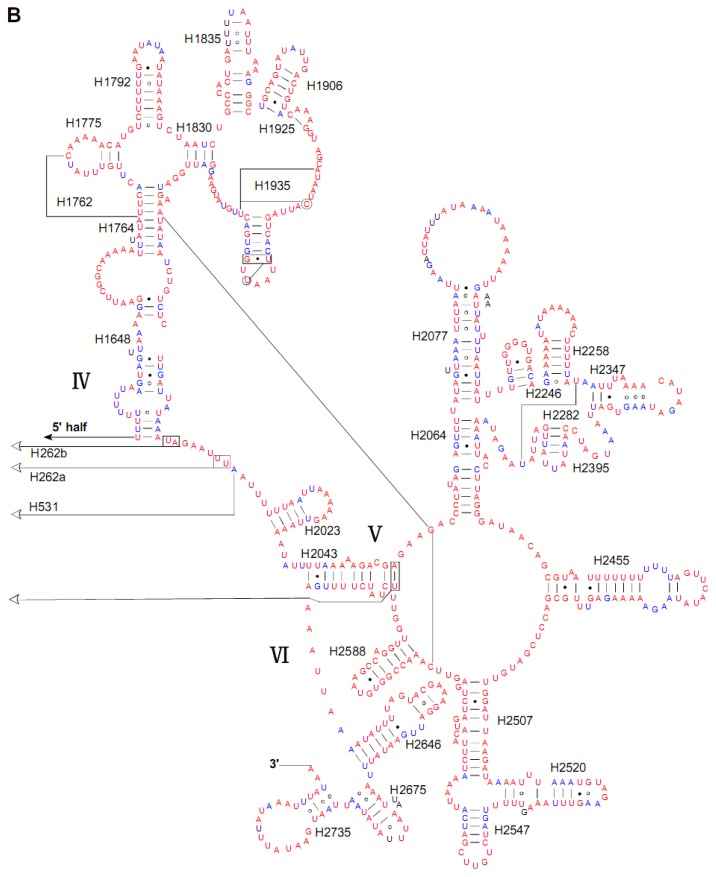
The predicted *rrnL* secondary structure from the *Sesamia inferens* mitogenome. The 5′ half of *rrnL* is displayed in **A** and the remaining 3′ half in **B**. Base-pairings are indicated as follows: Watson-Crick pairs by lines, wobble GU pairs by dots and mismatched pairs by circles.

**Figure 6:**
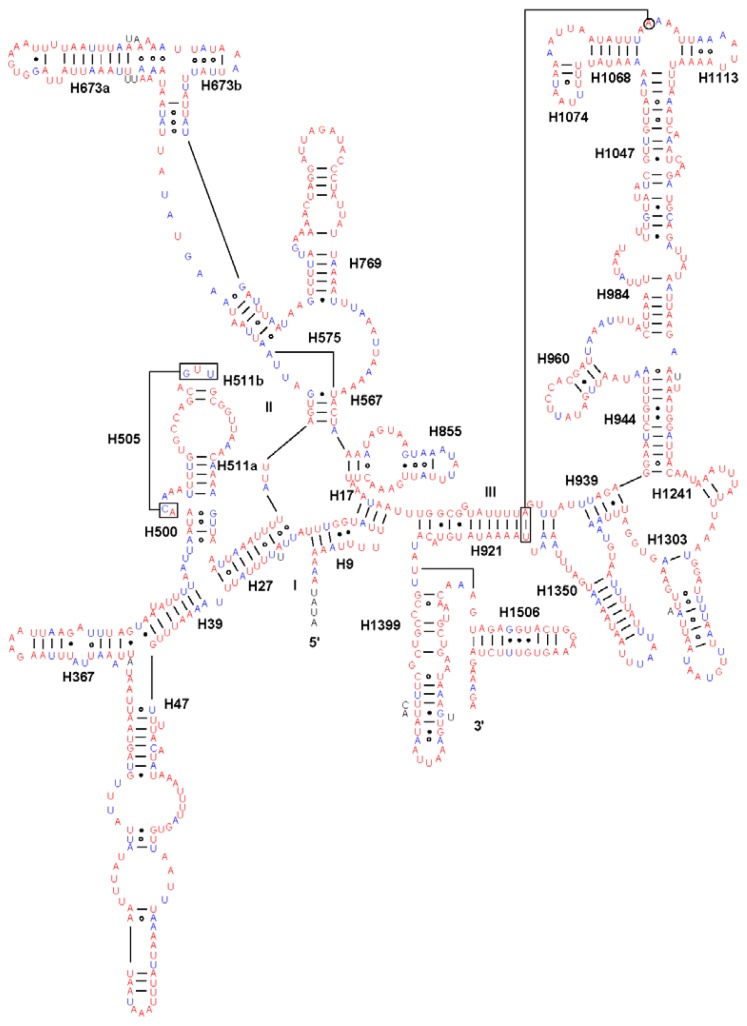
The predicted *rrnS* secondary structure in the *Sesamia inferens* mitogenome. Tertiary interactions and base triples are shown connected by continuous lines. Base-pairings are indicated in Figure 5.

Both the *rrnL* and *rrnS* secondary structures were predicted according to models proposed for these genes in other insects [27,28]. Although the length of individual helices varied in insect species, the secondary structures of both *rrnL* and *rrnS* in *S. inferens* were quite similar to their counterparts in *Apis mellifera*, *Manduca sexta*, and other insect species [27,28]. In *S. inferens*, the *rrnL* gene contained five domains (labeled I, II, IV, V, and VI) with 49 helices, as observed in other noctuid moths (Figure 5). Each domain was separated by a single-stranded region, and domain III was absent in arthropod mitogenomes. Also, *S. inferens* had the *rrnS* with 33 helices in three domains (labeled I, II, III) (Figure 6).

### 2.5. Non-Coding and Overlapping Regions

**Figure 7:**
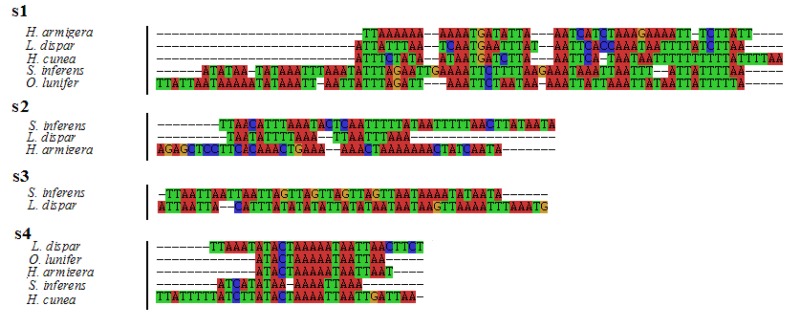
Intergenic spacer sequences in the mitogenomes of *Sesamia inferens* and other noctuid moths.

In the *S. inferens* mitogenome, there was a total of 257 bp of intergenic spacer sequences, which resided at 18 locations (except for the A+T-rich region) and varied in size from 1 to 68 bp. It is to be noted that there were four major intergenic spacers (s1–s4) spanning at least 18 bp, all of which were rich in As and Ts (Figure 7). The s1 spacer (68 bp) located between *trnQ* and *nad2* had a higher AT content compared with the counterpart in the *O. lunifer* mitogenome. And so did the other three spacers. The s3 spacer (44 bp), inserted between *nad6* and *cob*, is not conserved in five noctuid moths, which was mostly a 4 bp repeat in the *S. inferens* mitogenome while the other had a stretch of TA dinucleotides in the *L. dispar* mitogenome. However, the s2 spacer (44 bp), placed between *nad4* and *nad4L*, was longer than its counterparts in *H. armigera* and *L. dispar* mitogenomes, but it was twice the length of the counterpart in the *L. dispar* mitogenome (44 *vs.* 22) and about the same size as the counterpart in the *H. armigera* mitogenome (44 *vs.* 43). In addition, the s4 spacer (18 bp), located between *trnS2(UCN)* and *nad1*, did not contain the motif “ATACTAA” that was conserved across four other noctuid moths and other lepidopteran species mitogenomes [28,29].

Also, a total of 25 bp overlapping nucleotides was scattered over six locations and ranged in sizes from 1 to 11 bp in the *S. inferens* mitogenome, with the longest one (11 bp) located between *atp8* and *atp6*. Interestingly, an 8-bp motif “AAGCCTTA” conserved in the four other noctuid moths’ mitogenomes [4–7] was also detected between *trnW* and *trnC* in the present study.

### 2.6. A+T-rich Region

In mitochondrial genomes, the A+T-rich region had been reported to possess essential elements involved in the initiation of replication and transcription of mitogenome [30]. The A+T-rich region of the *S. inferens* mitogenome was 311 bp in length with an AT content of 95.8%. Similarly, the sizes and AT contents in the A+T-rich regions of four other noctuid moths were 328 bp and 95.1% AT content (*H. armigera*), 357 bp and 95.0% AT content (*H. cunea)*, 369 bp and 96.1% AT content (*L. dispar)*, and 319 bp and 93.4% AT content (*O. lunifer*) [4–7]. Notably, the *S. inferens* A+T-rich region displayed a higher AT content (95.8%) than all the other locations in the mitogenome (Table 2).

**Figure 8:**
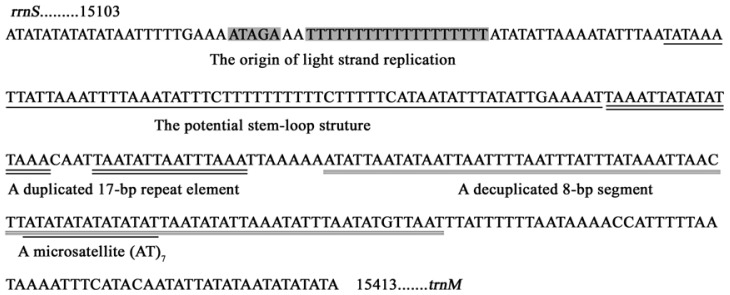
The structure of the A+T-rich region of the *Sesamia inferens* mitogenome. The origin of light strand replication, the potential stem loop structure, the duplicated 17-bp repeat element, the decuplicated 8 bp segment (derived from a consensus sequence “ATATTAAT”) and the microsatellite “(AT)*_n_*” elements are displayed as indicated in the figure.

**Figure 9:**
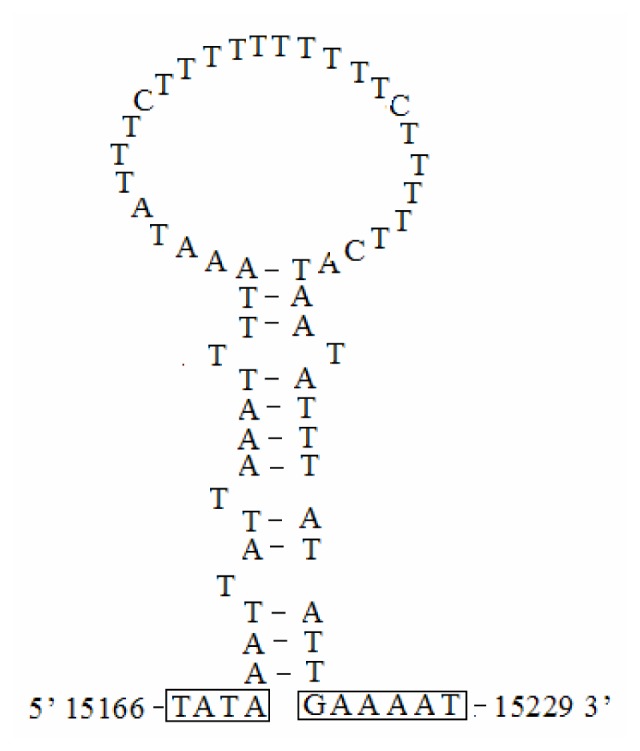
The potential stem-loop structure found in the *Sesamia inferens* A+T-rich region. The motifs (“GAAAAT” and “TATA”) in the flanking region of the stem-loop structures are indicated by boxing [32].

There was a conserved structure that combined the motif “ATAGA” and a 19-bp poly-T stretch in *S. inferens* A+T-rich region (Figure 8). A very similar pattern occurred in four other noctuid moths, and it was widely conserved in lepidopteran mitogenomes and might be the origin of light-strand replication [4–7,30]. There was also a stem-and-loop structure in *S. inferens* A+T-rich region containing a 5′ flanking “TATA” and 3′ flanking “G(A)*_n_*T” sequence, which were considered to be the site of initiation of secondary strand synthesis [31,32] (Figures 8 and 9). The stem-and loop structure might be the characteristic of insect mitogenomes, and it had been found in several insect orders including Orthoptera, Lepidoptera, Diptera, Plecoptera, and Hymenoptera, but it was not observed in four other noctuid moths [30,33–36]. Also, the conserved 3′ flanking “G(A)*_n_*T” sequence might not be present in all insects or the sequence might take on different forms [35,37].

The presence of varying copy numbers of tandemly repeated elements was one of the characteristics in insect mitogenomes [30]. The *S. inferens* A+T-rich region contained a duplicated 17-bp repeat element with minor variations, and a decuplicated highly divergent 8-bp segment “ATATTAAT” (Figure 8), which resembled the octuplicated 8-bp repeat element “TATATATT” in the *L. dispar* A+T-rich region [7]. Additionally, there was a microsatellite “(AT)_7_” in *S. inferens* A+T-rich region (Figure 8), and similar patterns were observed in *H. cunea* “(AT)_8_” and *O. lunifer* “(AT)_7_” mitogenomes [4,5].

A conserved 9-bp poly-A element was observed upstream of *trnM* in *H. cunea*, *L. dispar*, and *O. lunifer* mitogenomes, which might be involved in controlling transcription and replication initiation [4,5,7,38]. However, this structure was not detected in *S. inferens* and *H. armigera* mitogenomes [6].

### 2.7. Phylogenetic Relationships

In this study, the amino acid sequences of the 13 PCGs were concatenated, rather than analyzed separately, to construct the phylogenetic relationships, which may result in a more complete analysis [39]. Based on morphological and genomic analyses, the relationship among six superfamilies in lepidopteran species has been reported in other articles [29,40,41].

**Figure 10:**
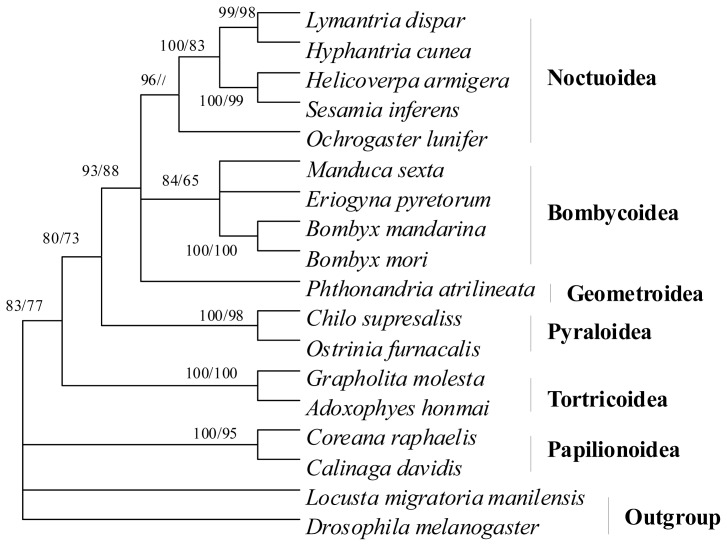
Phylogeny of the lepidopteran species. Phylogenetic tree inferred from amino acid sequences of 13 PCGs of the mitogenome by using Neighbor Joining (NJ) and Maximum parsimony (MP). Numbers at each node indicate bootstrap support; percentages of NJ probabilities (first value) and MP bootstrap support values (second value), respectively. *Drosophila melanogaster* [47] and *Locusta migratoria manilensis* were used as outgroup.

In the present study, the NJ and MP trees showed similar topologies except for some node confidence values (Figure 10). The *S. inferens* mitogenome together with mitogenomes of *H. armigera*, *H. cunea*, *L. dispar*, and *O. lunifer* clustered with other superfamilies, which was consistent with the morphological classification [40]. Geometroidea (*Phthonandria atrilineata*) was closely related to Noctuoidea and Bombycoidea (*Bombyx mandarina*, *Bombyx mori*, *Eriogyna pyretorum*, *M. sexta*) [28,42–44], but Papilionoidea (*Calinaga davidis* and *Coreana raphaelis*) [15,45] was the sister to Pyraloidea (*C. suppressalis* and *Ostrinia furnacalis*) [29,46], Tortricoidea (*Adoxophyes honmai* and *Grapholita molesta*) [16,38] and other superfamilies, in disagreement with the tree topology [29,40,41]. The results indicated that more mitogenomes could elaborate on phylogenetic relationships among lepidopteran superfamilies in greater detail to some extent.

## 3. Experimental Section

### 3.1. DNA Extraction

Adult *S. inferens* were collected from rice paddies surrounding Yangzhou (32°40.025N, 119°44.017E), Jiangsu Province, China. Samples were preserved in 100% ethanol and stored at −70 °C until DNA extraction was performed. Whole genomic DNA was extracted from a single sample using the protocol of DNAVzol (Bioteke, Beijing, China) and then used for the PCR amplification.

### 3.2. Primers Design, PCR Amplification, Cloning and Sequencing

Two pairs of LA-PCR primers were used for amplification of two overlapping fragments of *S. inferens* mitogenome, which were 16SAA (5′-ATGCTWCCTTTGCACRGTCAAGATACYGCGGC-3′), 16SBB (5′-CTTATCGAYAAAAAAGWTTGCGACCTCRATGTTG-3′), LP01 (5′-TGATTAGCTCCACAAATTTCTGAACATTGACC-3′), and LP02 (5′-WACACCAGTTCATATTDAACCAGAATGATATT-3′) [48]. Sub-PCR primers were designed from comparisons of 35 lepidopteran sequences available in GenBank and referenced to the universal primers of insect mitogenomes [49].

The conditions for amplification of two long fragments were as follows: An initial denaturation for 2 min at 96 °C followed by 35 cycles of 10 s at 98 °C, 10 min at 58 °C, and a subsequent 10 min final extension at 72 °C. PCR conditions for amplification of other fragments were as follows: An initial denaturation for 5 min at 95 °C, followed by 35 cycles of denaturation for 1 min at 94 °C, annealing for 1 min at 45–50 °C, elongation for 1–3 min (depending on putative length of the fragments) at 68 °C, and a final extension step of 72 °C for 10 min. For most fragments, LA Taq polymerase (TaKaRa, Kyoto, Japan) was used for the PCR amplification, but for fragments less than 1.3 kb, LA Taq polymerase was replaced by Taq polymerase (TaKaRa, Kyoto, Japan) in the PCR reaction. All PCR reactions were performed in an ABI thermal cycler (PE Applied Biosystems, CA, USA).

PCR products were separated by electrophoresis in a 1.0% agarose gel and purified using a DNA Gel Extraction Kit (Bioteke, Beijing, China). Purified PCR products were ligated into the T-vector (TaKaRa, Kyoto, Japan) and then transformed into XL-1 blue competent bacteria, according to the method of Wei *et al*. [50]. The positive recombinant clone was sequenced using upstream and downstream primers from bi-directions on ABI 3730XL Automated DNA Sequencer (PE Applied Biosystems, CA, USA) at least three times.

### 3.3. Sequence Analysis

The Staden package was used for sequence assembly and annotation [51]. The PCGs and rRNA genes in *S. inferens* mitogenome were identified by sequence alignment with other lepidopteran species, especially the Noctuidae by using Clustal X version 2.0 [4–7,52]. The PCG nucleotide sequences without start and termination codons were translated on the basis of the Invertebrate Mitochondrial Genetic Code. The AT content was calculated using MEGA version 5.0 [35]. Composition skew analysis was carried out with formulas AT skew = [A − T]/[A + T] and GC skew = [G − C]/[G + C] [53].

Transfer RNA genes were identified using tRNAscan-SE software available online [54], and the software of XRNA 1.2.0b was used to draw the secondary structure of RNA genes. The secondary structures of the *rrnL* and *rrnS* genes were inferred based on models developed for other insect species [27,55]. To infer the rRNA gene secondary structures, we used a commonly accepted comparative approach to correct for unusual pairings with RNA-editing mechanisms that are well known in arthropod mitogenomes [56,57]. The entire A+T-rich region was subjected to a search for tandem repeats using the Tandem Repeats Finder program [58].

### 3.4. Phylogenetic Analysis

To illustrate the phylogenetic relationship of Lepidoptera, the other sixteen complete lepidopteran mitogenomes were downloaded from Genbank, including *A. honmai* (DQ073916), *B. mori* (AY048187), *B. mandarina* (FJ384796), *C. davidis* (HQ658143), *C. raphaelis* (DQ102703), *C. suppressalis* (JF339041), *E. pyretorum* (FJ685653), *G. molesta* (HQ392511), *H. armigera* (GU188273), *H. cunea* (GU592049), *L. dispar* (FJ617240), *M. sexta* (EU286785), *O. furnacalis* (AF467260), *O. lunifer* (AM946601), *P. atrilineata* (EU569764), *Drosophila melanogaster* (U37541) and *Locusta migratoria manilensis* (GU344101) were used as outgroup.

The alignment of the concatenated amino acid sequences of each 13 mitochondrial PCGs was aligned with Clustal X [52] using default settings. Then, based on the concatenated amino acid data set from the 13 PCGs, phylogenetic analyses were constructed using MEGA version 5.0 software with Neighbor Joining (NJ) and Maximum Parsimony (MP) methods [35]. Bootstrap analysis was done with 1000 replications, and bootstrap values were calculated using the 50% majority rule.

## 4. Conclusions

In the present study, the mitogenome for *S. inferens* was one of five sequenced mitogenomes of the Noctuidae species, which includes about 10% of all recorded lepidopteran species. Compared to four other noctuid moths, the newly determined mitogenome also shared the gene order, nucleotide composition of protein-coding genes, the presence of intergenic spacers, and other features [4–7]. The order of *trnM*, *trnI*, and *trnQ* in the mitogenome might be one of the features that characterize the whole order Lepidoptera. The *S. inferens* mitogenome was biased to use T rather than A, which was different from other noctuid moths. The s4 spacer did not contain the motif “ATACTAA” that was one potential characteristic feature conserved across four other noctuid moths, the reason may be related to genetic mutation [59]. The great difference was that the stem-and-loop structure was found in the A+T-rich region of *S. inferens* mitogenome, but it was not observed in the four other noctuid moths. Although the *S. inferens* mitogenome was added, the position of Noctuidae is not improved. More insect mitogenomes will be helpful to fully resolve the phylogeny of Noctuoidea.

## Figures and Tables

**Table 1:** Annotation of the mitogenome of *Sesamia inferens*.

Name	Direction	Site	Size (bp)	Intergenic spacer	Anticodon	Start codon	Stop codon
*trnM*	F	1..68	68	0	32..34 CAT		
*trnI*	F	69..133	65	−3	98..100 GAT		
*trnQ*	R	131..199	69	68	167..169 TTG		
*nad2*	F	268..1284	1017	6		ATT	TAA
*trnW*	F	1291..1358	68	−8	1322..1324 TCA		
*trnC*	R	1351..1423	73	4	1391..1393 GCA		
*trnY*	R	1428..1494	67	5	1460..1462 GTA		
*cox1*	F	1500..3030	1531	3		CGA	T-
*trnL2(UUR)*	F	3034..3100	67	0	3064..3066 TAA		
*cox2*	F	3101..3782	682	0		ATT	T-
*trnK*	F	3783..3853	71	0	3813..3815 CTT		
*trnD*	F	3854..3919	66	0	3884..3886 GTC		
*atp8*	F	3920..4087	168	−11		ATT	TAA
*atp6*	F	4077..4754	696	−1		ATG	TAA
*cox3*	F	4754..5542	789	2		ATG	TAA
*trnG*	F	5545..5609	65	0	5575..5577 TCC		
*nad3*	F	5610..5963	354	16		ATT	TAA
*trnA^)^*	F	5980..6048	69	2	6010..6012 TGC		
*trnR*	F	6051..6114	64	9	6078..6080 TCG		
*trnN*	F	6124..6188	65	2	6154..6156 GTT		
*trnS1(AGN)*	F	6191..6256	66	0	6216..6218 GCT		
*trnE*	F	6257..6324	68	8	6287..6289 TTC		
*trnF*	R	6333..6399	67	−1	6364..6366 GAA		
*nad5*	R	6399..8150	1752	0		ATT	TAA
*trnH*	R	8151..8215	65	0	8183..8185 GTG		
*nad4*	R	8216..9554	1339	44		ATG	T-
*nad4L*	R	9599..9892	294	7		ATG	TAA
*trnT*	F	9900..9969	70	0	9929..9931 TGT		
*trnP*	R	9970..10034	65	7	10002..10004 TGG		
*nad6*	F	10042..10575	534	44		ATC	TAA
*cob*	F	10620..11768	1149	1		ATG	TAA
*trnS2(UCN)*	F	11770..11836	67	18	11802..11804 TGA		
*nad1*	R	11855..12799	945	1		ATG	TAA
*trnL1(CUN)*	R	12801..12867	67	−1	12836..12838 TAG		
*rrnL*	R	12867..14251	1385	0	-		
*trnV*	R	14253..14318	66	0	14284..14286 TAC		
*rrnS*	R	14319..15102	784	0			
A+T-rich region		15103..15413	311	0			

* Negative numbers indicate that adjacent genes overlap.

**Table 2:** Skewed nucleotide composition in regions of noctuid moths’ mitogenomes.

Region	Whole mitogenome		PCGs				
		
Species	Length (bp)	AT (%)	Length (bp)	AT (%)	1st position (%)	2nd position (%)	3rd position (%)
*H. armigera*	15347	81.0	11,133	79.4	70.2	94.1	73.8
*H. cunea*	15481	80.4	11,136	78.5	73.2	70.3	92.0
*L. dispar*	15569	79.9	11,169	77.7	90.2	72.9	70.1
*O. lunifer*	15593	77.8	11,196	75.7	72.0	70.0	85.1
*S. inferens*	15413	80.3	11,160	78.6	87.7	72.9	75.2
